# Supplementary material for: Venomix: a simple bioinformatic pipeline for identifying and characterizing toxin gene candidates from transcriptomic data
Source: PeerJ. 2018 Jul 31;6:e5361. doi: 10.7717/peerj.5361 (PMC6074769; doi:10.7717/peerj.5361)
Supplement: Supplemental Information 6 [file peerj-06-5361-s006.gz › FinalOutput_1E-6/Cysteine-rich_venom_protein_Mr30_2/finaltree.pdf]

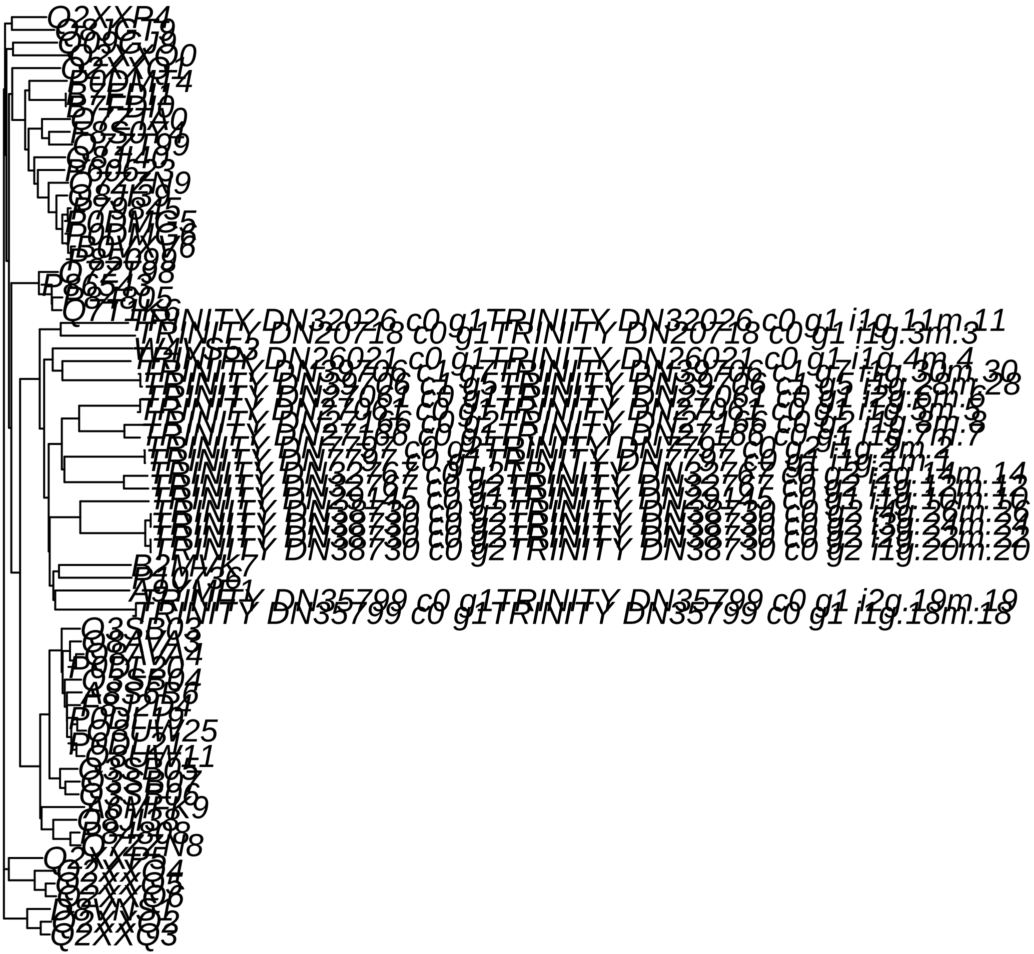
Q2XXP4
TRINITY DN32026 c0 g1TRINITY DN32026 c0 g1 i1g.11m.11
TRINITY DN20718 c0 g1TRINITY DN20718 c0 g1 i1g.3m.3
TRINITY DN38730 c0 g2TRINITY DN38730 c0 g2 i1g.20m.20
B2MVK7
TRINITY DN35799 c0 g1TRINITY DN35799 c0 g1 i2g.19m.19
TRINITY DN35799 c0 g1TRINITY DN35799 c0 g1 i1g.18m.18
Q2XXQ3
